# BindFlow: A Free, User-Friendly Pipeline for Absolute Binding Free Energy Calculations Using Free Energy Perturbation or MM(PB/GB)SA

**DOI:** 10.1021/acs.jctc.5c02026

**Published:** 2026-01-07

**Authors:** Alejandro Martínez León, Lucas Andersen, Jochen S. Hub

**Affiliations:** Theoretical Physics and Center for Biophysics, Universität des Saarlandes, 66123 Saarbrücken, Germany

## Abstract

We present BindFlow, a Python-based software for automated absolute binding free energy (ABFE) calculations at the free energy perturbation (FEP) or at the molecular mechanics Poisson–Boltzmann/generalized Born surface area [MM­(PB/GB)­SA] level of theory. BindFlow is free, open-source, user-friendly, and easily customizable, runs on workstations or distributed computing platforms, and provides extensive documentation and tutorials. BindFlow uses GROMACS as a molecular dynamics engine and provides built-in support for the small-molecule force fields GAFF, OpenFF, and Espaloma, as well as support for user-provided custom force fields. We test BindFlow by computing affinities for 139 receptor–ligand pairs, involving eight different targets, including six soluble proteins, one membrane protein, and one nonprotein host–guest system. We find that the agreement of BindFlow predictions with experiments is overall similar to gold standards in the field. Interestingly, we find that MM­(PB/GB)­SA achieves correlations that, for some systems and force fields, approach those obtained with FEP while requiring only a fraction of the computational cost. This study establishes BindFlow as a validated and accessible tool for ABFE calculations.

## Graphical abstract

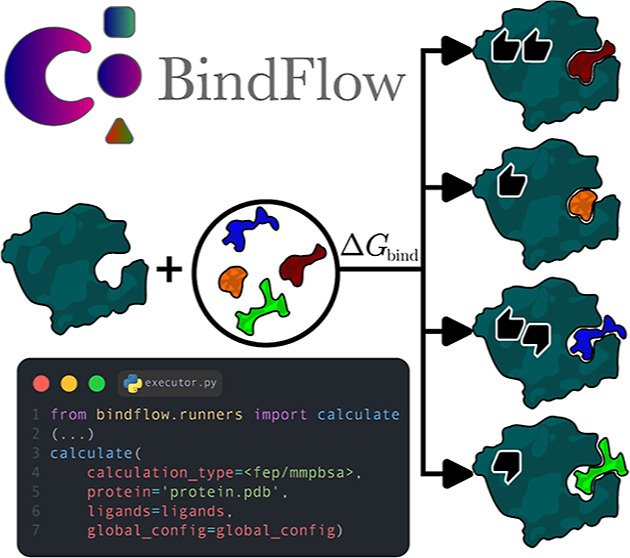

## Introduction

During the early stages of drug discovery, a large number of chemical compounds are evaluated with the aim of finding potent binders, a process that is both time-consuming and resource-intensive.^1,2^ Thus, computational models are widely used to estimate the binding free energy of small molecules to biological targets in order to prioritize compounds for follow-up synthesis and experimental evaluation.^3–6^ In the 1950s, methods collectively known as alchemical free energy perturbation were introduced.^7^ The term “free energy perturbation” (FEP) referred originally to a specific class of alchemical methods^8^ but has since been more generally applied to alchemical binding free energy methods, a notion followed in this study. FEP enabled the estimation of free energy differences at a fraction of the computational cost compared with conventional molecular dynamics (MD) simulations. Nevertheless, FEP remained computationally demanding for high-throughput studies, rationalizing the development of computationally more efficient yet more approximate end-point free energy techniques such as the molecular mechanics Poisson–Boltzmann/generalized Born surface area [MM­(PB/GB)­SA] methods,^9^ which achieved varying degrees of success.^10^ Recent advances in biomolecular simulation methods^6,11^ combined with the growth of computational power have enabled increasingly accurate affinity predictions,^6,12^ making both FEP and MM­(PB/GB)­SA calculations more routine.

Binding free energy estimation has been categorized into relative (RBFE) and absolute binding free energy (ABFE) calculations.^11^ RBFE is typically preferred for ranking congeneric molecular series^13^ or for evaluating the effects of mutations on ligand binding.^14^ Alchemical RBFE methodologies have seen substantial advancements, resulting in numerous implementations.^15–29^ These developments have enabled alchemical RBFE methods to achieve remarkable accuracy, often with a root mean-squared error (RMSE) of 1–2 kcal mol^–1^ relative to experimental values,^30^ making them highly effective for drug discovery applications.^13–15,31–38^

ABFE calculations yield the binding free energy relative to a standard state of the compound in solution.^39^ ABFE calculations are particularly useful for studying highly diverse molecular sets,^4,40–42^ for binding pose validation,^43,44^ or for multitarget selectivity prediction.^45,46^ Alchemical ABFE methods have demonstrated their power in addressing complex challenges across various projects,^4,34,40,41,45–49^ and large-scale comparisons with experimental data have revealed that alchemical ABFE predictions are approaching experimental accuracy.^50^

However, the preparation, execution, and analysis of ABFE simulations are tedious, system-dependent processes that frequently rely on expert intervention. Fully automating the binding free energy pipeline can mitigate these challenges, enabling high-throughput calculations for drug discovery campaigns while improving reproducibility and accessibility. We suggest that a robust and accessible ABFE workflow should meet six properties: (i) open-source commitment to ensure transparency and enable community-driven method and code development; (ii) efficiency by a flexible and error-tolerant use of computational resources, in particular in distributed platforms such as high-performance computing (HPC) clusters; (iii) reliability, as demonstrated through extensive benchmarking across sets of diverse ligands and receptors, including challenging targets; (iv) flexibility for expert users to test effects of using different force fields or diverse simulation and deployment parameters; (v) multiligand end-to-end automation, reducing human error and setup time and increasing reproducibility; and (vi) accessibility, supported by comprehensive documentation and tutorials. While several powerful ABFE workflows have been established, they typically emphasize only a subset of these properties.

Commercial solutions such as FEP+^47^ have set high standards for reliability and automation in the pharmaceutical industry,^47^ and other commercial platforms like XFEP^17^ have contributed to broader adoption. However, their closed-source design and licensing costs restrict accessibility for broad academic use and do not support the methodological development by the scientific community. In contrast, academic and open-source tools^23,29,51–61^ have played a key role in democratizing ABFE calculations and expanding the ecosystem of automated free energy workflows. However, available academic tools are limited to diverse aspects. Certain tools have been validated using rather small receptor–ligand data sets or provide limited or even nonexisting documentation or tutorials. Other tools automate only part of the workflow, such as the setup of a simulation system and topologies but not the deployment to distributed computing environments or data analysis. Certain tools are focused on specific force fields, use a nonfree MD engine, or support limited options for expert users for customizing MD parameters or the λ-schedule. A brief overview of available tools is provided in the Supporting Discussion. In summary, to the best of our knowledge, no available software for ABFE calculations commits to all six properties outlined above.

Here, we aim to close this gap by introducing *BindFlow*, a free, user-friendly, open-source software package that provides a multiligand, end-to-end automated ABFE workflow and offers fine-grained control over simulation and deployment parameters (Figure 1a). BindFlow implements two ABFE methods: (i) the end-point free energy method MM­(PB/GB)­SA,^9^ based on a simulation of the receptor–ligand complex only (also referred to as the single-trajectory approach), and (ii) a double-decoupling alchemical free energy method that uses thermodynamic integration (TI)^62^ or the multistate Bennett acceptance ratio (MBAR)^63^ frameworks for free energy estimation, following Alibay et al.^5^ and Ries et al.^52^ BindFlow uses GROMACS as the MD engine. BindFlow is highly user-friendly and provides extensive tutorials and documentation. It natively supports the small-molecule force fields GAFF, OpenFF, and Espaloma, as well as any GROMACS-compatible force field. At present, it schedules tasks on either a local desktop or SLURM-based distributed computing environments while also providing scope for incorporating additional HPC platforms in the future. By scheduling its tasks with Snakemake,^64^ BindFlow efficiently uses the available hardware and is resilient against rare hardware failures or simulation instabilities. For advanced users, this allows full control over the pipeline and MD settings and provides various options for further customization. BindFlow has been forked from *ABFE_workflow* ^52^ but resolves its technical restrictions and introduces diverse new functionalities and numerous options for customization.

**Figure 1:**
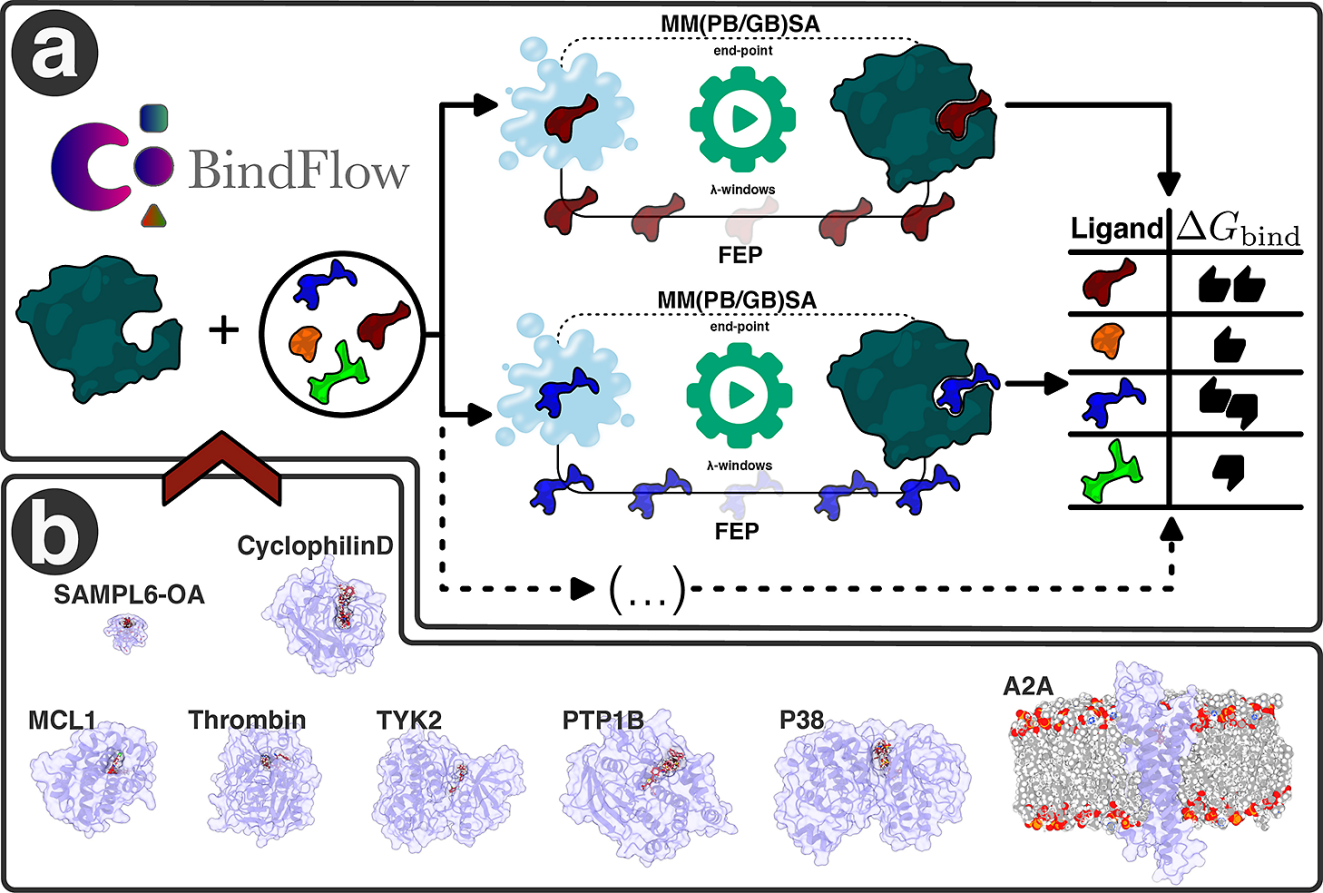
(a) Schematic representation of the BindFlow workflow and (b) the systems used in this study for validating ABFE calculations.

Below, we first present implementation principles and Application Programming Interface (API) concepts of BindFlow. Next, we validate BindFlow by computing ABFEs for 139 diverse ligands binding to eight different receptors, including six soluble proteins, one membrane protein, and one host–guest system (Figure 1b). The calculations include both FEP and MM­(PB/GB)­SA methods using three widely used open-source small-molecule force fields: GAFF-2.11,^65^ OpenFF-2.0.0,^66^ and Espaloma-0.3.1,^67^ referred to as GAFF, OpenFF, and Espaloma in the following. Statistical uncertainties are rigorously assessed by running at least three independent replicates for each free energy calculation workflow.

Overall, our results show that BindFlow delivers a predictive performance on par with established gold standards in the field. Strikingly, we observe that for certain targets and force fields, the computationally efficient MM­(PB/GB)­SA method achieves correlations with the experiment that approach those of the more rigorous FEP calculations—yet at only a fraction of the computational cost. By fully automating the binding free energy workflow and minimizing user intervention, BindFlow enhances not only efficiency but also reproducibility. In addition, BindFlow scales efficiently across HPC environments, enabling large simulation campaigns to be executed with high throughput and resource utilization. Together, these features make BindFlow a practical, accessible, and reliable platform for large-scale binding affinity prediction with strong potential for accelerating modern drug discovery campaigns.

## Theory

The theories of FEP and MM­(PB/GB)­SA have been reviewed frequently;^9,10,12,63,68–70^ hence, we provide only a brief summary of the underlying concepts.

### MM­(PB/GB)­SA

Following the thermodynamic cycle in Figure 2a, MM­(PB/GB)­SA estimates the binding free energy Δ*G*_bind_ as follows^9,10,69,70^

1 $$\begin{array}{ll} \Delta G_{bind} & =-\left(\Delta G_{S}^{R}+\Delta G_{S}^{L}\right)+\Delta G_{{bind}_{v}}+\Delta G_{S}^{RL}\\ \Delta G_{{bind}_{v}} & =G_{v}^{RL}-G_{v}^{R}-G_{v}^{L} \end{array}$$

Here, Δ*G*_S_^X^ denotes the solvation free energy for species X (X = R, L, or RL), where superscripts R, L, and RL denote the receptor, ligand, and receptor–ligand complex, respectively. The solvation free energy for species X is decomposed into polar and nonpolar terms, Δ*G*_S_^X^ = Δ*G*_PB/GB_^X^ + Δ*G*_SA_^X^. Δ*G*_PB/GB_^X^ is obtained by solving the Poisson–Boltzmann (PB) equation or using the generalized Born (GB) model,^10^ while Δ*G*_SA_^X^ is derived from the solvent-accessible surface area (SASA).^71^

**Figure 2:**
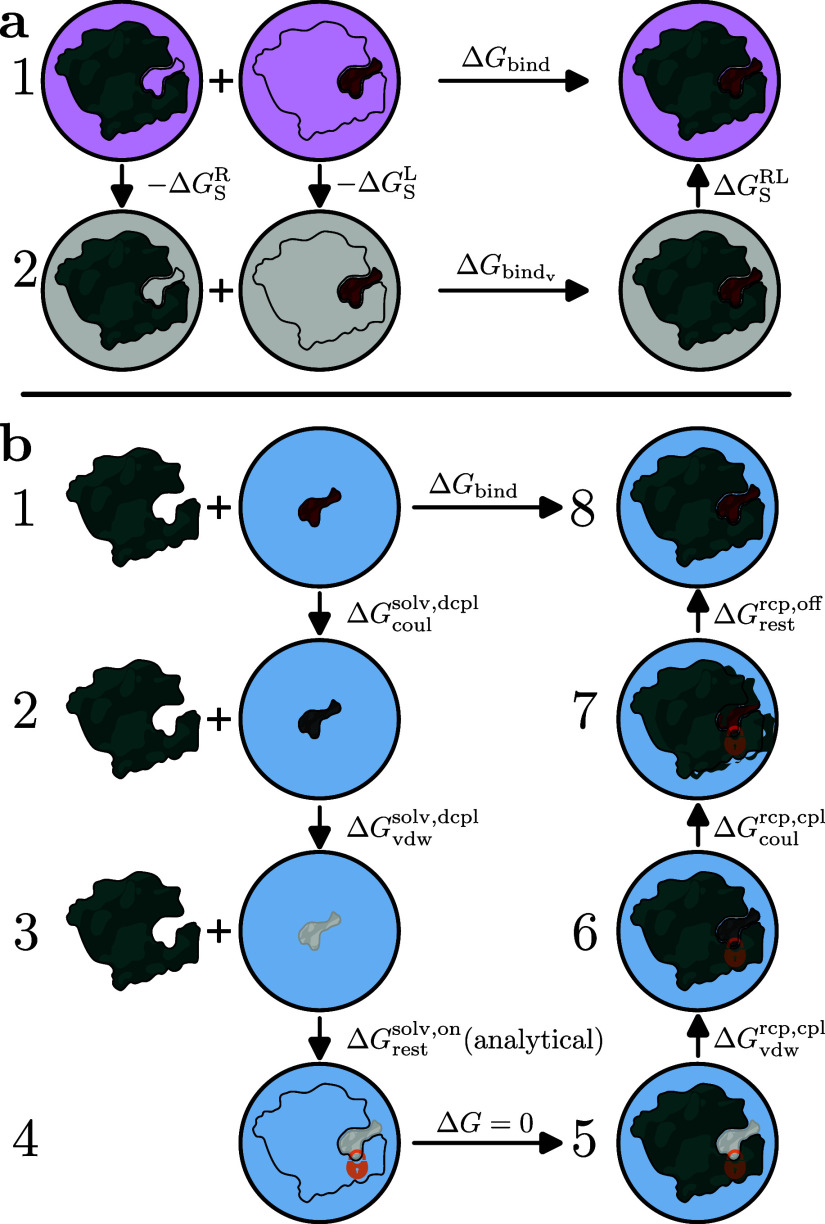
Thermodynamic cycles for (a) MM­(PB/GB)­SA and (b) FEP. Δ*G*_S_^X^ denotes the solvation free energy for species X, where superscripts R, L, and RL denote the receptor, ligand, and receptor–ligand complex, respectively. Δ*G*_bind_v__ is the binding free energy in vacuum. Superscripts “solv” and “rcp” indicate states with the ligand in solvent or in the receptor, respectively. Superscripts “dcpl” and “cpl” indicate decoupling or coupling processes, while “coul” and “vdw” specify transitions of Coulomb or Lennard-Jones interactions, respectively. Superscripts “on/off” specify the activation or deactivation of Boresch restraints (subscript “rest”). The light pink, gray, and light blue backgrounds represent the implicit solvation model, vacuum, and explicit water model, respectively.

Δ*G*_bind_v__ denotes the binding free energy in vacuum, computed from the free energies *G*_v_^X^ of the receptor, ligand, or receptor–ligand complex. The free energies are estimated via

2 $$G_{v}^{X}=E_{MM}^{X}-TS^{X}$$

where *E*_MM_^X^ is the molecular mechanics (MM) potential energy of species X comprising bonded and nonbonded terms, as defined by the MM force field. *T* is the temperature and *S* ^X^ is the entropy of species X. By defining the free energy of species X as

3 $$G^{X}=E_{MM}^{X}+\Delta G_{PB/GB}^{X}+\Delta G_{SA}^{X}-TS^{X}$$

Δ*G*_bind_ may be written as the free energy difference between products and reactants

$$\Delta G_{bind}=G^{RL}-G^{R}-G^{L}$$

thereby rationalizing by the subscripts of eq 3 the name MM­(PB/GB)­SA.^9,10,69,70^

BindFlow computes the energy contributions to Δ*G*_bind_ by averaging over multiple MD simulation frames using the package gmx_MMPBSA.^69^ The entropic term may be estimated using normal-mode analysis, interaction entropy (IE),^72^ or a cumulant approximation to the second order of the exponential average (C2).^73–75^ These entropy estimations have been implemented by gmx_MMPBSA^69^ and may therefore be used by BindFlow.

BindFlow employs a single-trajectory protocol; that is, all contributions from the ligand, receptor, or receptor–ligand complex are computed from MD simulations of the complex. This approach reduces the computational cost and facilitates the cancellation of bonded terms in *E*_MM_^X^. However, the approach neglects contributions from structural changes of the ligand or receptor upon binding.^10,69^

### FEP

Alchemical FEP estimates binding free energies by constructing a thermodynamic cycle. Such cycles involve decoupling the ligand from the solvent (Figure 2b, Steps 1–3) and coupling it back in the binding site (Figure 2b, Steps 5–7) in the presence of restraints. The restraints maintain the ligand in the binding pocket, thereby improving sampling and unambiguously defining the reference standard state. BindFlow employs Boresch restraints.^39^ The free energy for activating Boresch restraints in the solvent is calculated analytically (Figure 2b, Steps 3–4) and the free energy for removing the restraints in the receptor environment is derived numerically (Figure 2b, Steps 7–8).

Free energy differences Δ*G* between two states (e.g., between states 2 and 1 in Figure 2b) are estimated from simulations across a series of intermediate alchemical states, specified by a parameter λ. BindFlow calculates Δ*G* values using either TI^68^ or the MBAR^63^ method. TI computes the Δ*G* values by integrating the mean force along the λ parameter^68^

4 $$\Delta G_{1\to 2}=\int_{\lambda_{1}}^{\lambda_{2}}{\left\langle \frac{\partial U\left(\lambda \right)}{\partial \lambda }\right\rangle }_{\lambda }d\lambda$$

where ⟨···⟩_λ_ denotes the ensemble average at a given value λ and *U*(λ) is the potential energy. In practice, the integral is solved numerically by using a finite number of λ points. MBAR estimates Δ*G* values using information from all of the alchemical states. MBAR requires solving the following set of equations self-consistently to obtain the reduced free energies *f*_*i*_ ^63^

5 $$e^{-f_{i}}=\sum_{j=1}^{M}\sum_{n=1}^{N_{j}}\frac{e^{-u_{i}\left(x_{j,n}\right)}}{\sum_{k=1}^{M}N_{k}e^{-u_{k}\left(x_{j,n}\right)+f_{k}}}$$

Here, *M* is the number of λ-states, *N*_*j*_ is the number of samples from λ-state *j*, and *u*_*i*_(x_*j*,*n*_) is the reduced potential energy of the *n*th sample x_*j*,*n*_ from the simulation of λ-state *j* evaluated with the energy function of λ-state *i*.

## Implementation

### General Concept of BindFlow

As illustrated in Figure 3, BindFlow streams the entire binding free energy pipeline, including building of the simulations systems, definition of force fields and MD parameters, careful multistep equilibration routines, launching of productions simulations, and analysis. For FEP calculations, specifically, production simulations include the definition of Boresch restraints, as well as the setup, equilibration, and launching of all λ-windows. The pipeline is organized into tasks with well-defined dependencies, which are deployed to the computing environment. The environment may be a desktop computer, a parallelized high-performance computer, or another distributed computing environment. Task scheduling is carried out by Snakemake,^64^ a robust task manager that has been widely used for defining complex bioinformatics analyses but has to our knowledge been less used by the MD community. Snakemake constructs a direct acyclic graph that specifies the dependencies among BindFlow tasks to be executed asynchronously, thereby optimally using the available hardware. BindFlow uses GROMACS^76^ as a molecular dynamics engine.

**Figure 3:**
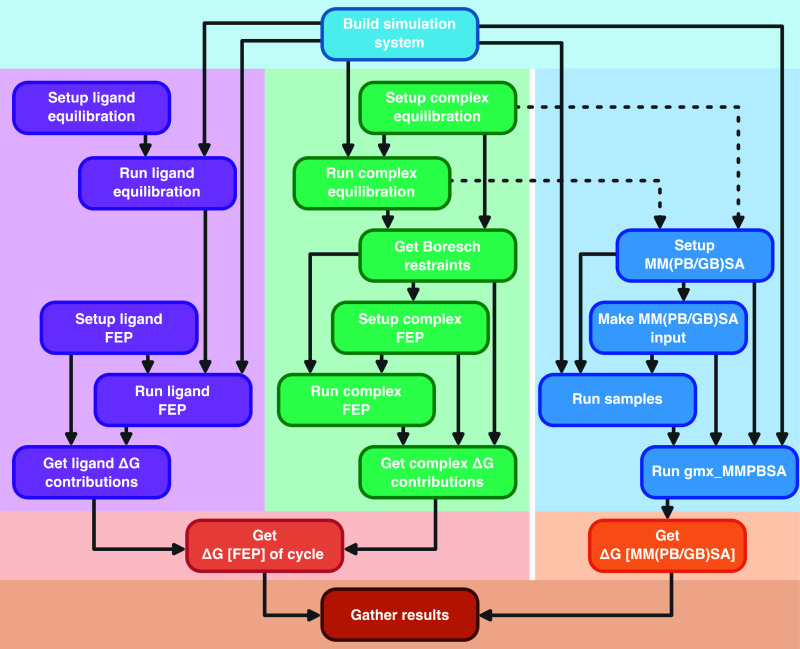
Task dependency graph for BindFlow. Each rectangle represents a task (or group of tasks), while arrows indicate dependencies between them. Some tasks are shared between methodologies. Purple boxes correspond to ligand-in-solvent FEP simulations, and green boxes correspond to receptor–ligand complex FEP simulations. The darkest blue boxes denote MM­(PB/GB)­SA simulations, which reuse some tasks from the receptor–ligand complex FEP workflow. All three approaches share the initial system building step (light blue) and converge at the final result gathering stage to report the binding free energy, Δ*G*_bind_.

BindFlow may compute Δ*G*_bind_ from a protein PDB file, together with a set of ligands provided as MOL files. However, for more complex systems including cofactors or membranes, additional input files may be provided.

The pipeline is implemented into a single function *calculate* within the module *runners*. The function *calculate* takes general parameters such as the protein and ligand structures and, through the keyword argument *global_config*, more specific definitions such as the computational environment or parameters for simulations analysis. Scheme 1 presents a minimalistic Python script for running an FEP pipeline in an HPC environment, while Listing S1 presents a more extended example for running an MM­(PB/GB)­SA pipeline on a desktop computer. Parameters passed with *global_config* may be specified within Python (Scheme 1) or, more conveniently, using JSON or YAML files (Listing S1). While BindFlow implements default workflows, the user has full control over equilibration and production settings, GROMACS parameters, and task deployment (Listing S7). Full documentation is provided online. Two classes for deployment are provided, namely, for a desktop computer and for the SLURM queuing system;^77^ however, alternative deployment managers may be added by the user via the abstract base class *Scheduler*, for instance, for using cloud computing services. Thus, BindFlow is highly user-friendly yet allows extensive customization by the user.

**Scheme 1:**
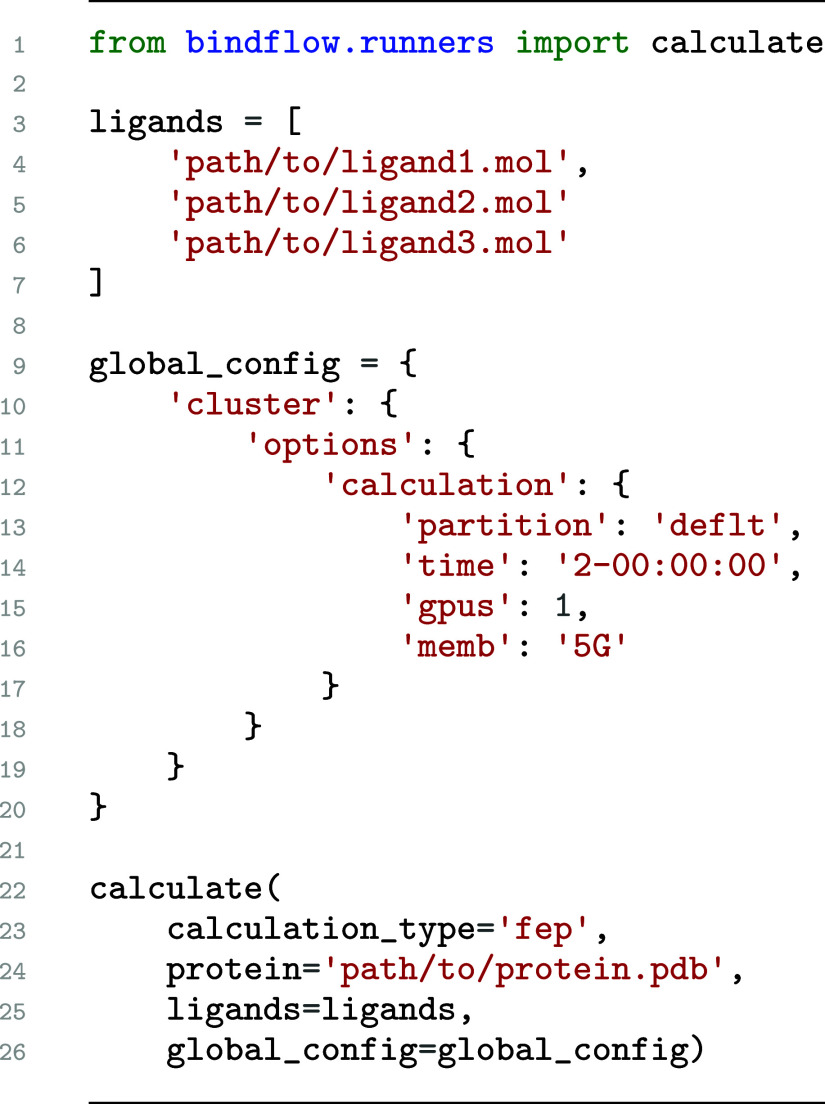
Minimal Python code for running an FEP campaign with BindFlow, as specified by *calculation_type*. The *protein* specifies the path to the protein PDB file, and the *ligands* specifies the list of ligand MOL files. The dictionary *global_config* specifies options for the computing environment.

BindFlow has been forked from ABFE_Workflow^52^ yet has been largely rewritten with the following aims in mind: options for extensive customization, efficient resource utilization, built-in support for several small-molecule force fields, flexibility with respect to user-provided force fields, comprehensive documentation, implementation of MM­(PB/GB)­SA, handling of cofactors, and support for membrane proteins and nonprotein receptors. BindFlow is released as an open source under the GPL-3.0 license.

### Input Files and Force Fields

For a typical workflow, the protein structure is provided as a PDB file and a set of ligands as MOL files, in which the ligand coordinates are aligned with the protein binding pocket. Optionally, cofactors are provided as MOL files. Running membrane protein systems requires additional care, as described in the online documentation. To enable the use of custom force fields or to simulate complex receptors, the user may provide GROMACS topology (TOP) and structure files (GRO), allowing BindFlow to operate with any GROMACS-compatible force field or simulation system. Examples for system component definitions are given in Listings S2, S3, S4, S5, and S6. More technical details are provided in the online documentation.

To achieve reliable ABFE results, accurate initial structural models are crucial with respect to the correct receptor–ligand arrangement and correct tautomeric, isomeric, and protonation states.^78^ While BindFlow does not aim to solve these upstream challenges, it provides basic tools for structural cleanup, for instance, for resolving missing atoms or correcting atom names, via the pdbfixer^79^ and pdb2gmx^76^ software tools.

By default, proteins are described with Amber99sb-ildn,^80^ membranes with SLipids2020,^81^ cofactors and ligands with OpenFF-2.0.0,^66^ and water molecules with the TIP3P model.^82^ Other protein force fields may be selected from the GROMACS distribution or from a user-specified path. Alternative water models can be chosen. The small-molecule force fields OpenFF,^66^ GAFF,^65^ and Espaloma^67^ are natively supported by BindFlow via the TOFF^83^ package. Because the choice of the force field may influence the accuracy of ABFE calculations, users are encouraged to consult the literature when selecting a force field for their system. For example, Hahn et al.^84^ recently reviewed small-molecule force fields in the context of FEP calculations.

## Results

### Validation Set Involving 139 Receptor–Ligand Pairs and Three Small-Molecule Force Fields

Comprehensive benchmarks are computationally costly, which explains their limited presence in academic ABFE workflows. In developing BindFlow, however, we placed particular emphasis on rigorously validating its FEP and MM­(PB/GB)­SA methodologies. To this end, we computed the binding affinity of 139 receptor–ligand pairs for which high-quality experimental data are available (Figure 1b). The receptors included six soluble proteins (cyclophilin D, MCL1, thrombin, TYK2, PTP1B, P38), the transmembrane GPCR protein A2A, and the nonprotein host–guest system SAMPL6-OA that has been used for a binding affinity prediction challenge.^86^ Affinity data for P38, PTP1B, TYK2, thrombin, and MCL1 have been widely utilized for validating RBFE^15–18^ and ABFE calculations,^17,47,87^ allowing us to compare BindFlow results with the literature (see below). These systems involve various challenges for binding affinity calculations. (i) Some ligands binding to PTP1B, thrombin, MCL1, or SAMPL6-OA are charged (Figures S18, S20, S21, S23). (ii) Ligands binding to PTP1B or P38 may proceed via slow induced-fit conformational transitions, suggesting that simulations may not sufficiency sample the apo state and, thereby, partly miss the free energy cost for the induced-fit transition.^47^ (iii) Binding affinities may be influenced by cofactors: for P38, PTP1B, TYK2, and thrombin, crystallographic water molecules may interact with the ligand (Table 1, sixth column). In A2A, a sodium ion interacts with the ligand, suggesting that imprecise modeling of a salt bridge by the force field may add considerable uncertainty. (iv) Among the ten ligands binding to cyclophilin D (Figure S22), four comprise more than 30 heavy atoms, which could potentially increase sampling challenges, while (v) eight ligands contain exotic ring structures, posing challenges for the accurate force field representation.^5,52,53^ Thus, our validation sets involve highly diverse receptor–ligand pairs that cover various common challenges during binding affinity calculations. All ligands are shown in Figures S16–S23.

**Table 1:** Simulation Systems Used for Validating BindFlow

System	Number of Atoms	Force Field		Number of Ligands	Cofactor (s)	Dynamic Range [kcal mol^–1^]	References
		Protein	Ligands				
P38	86,376	Amber14sb	Espaloma-0.3.1	29	3H_2_O	3.80	Hahn et al.^30^
			GAFF-2.11				
			OpenFF-2.0.0				
A2A	84,996	Amber14sb	Espaloma-0.3.1	10	Na^+^	2.69	Deflorian et al.^13^
			GAFF-2.11				
			OpenFF-2.0.0				
PTP1B	74,246	Amber14sb	Espaloma-0.3.1	22	4H_2_O	5.17	Hahn et al.^30^
			GAFF-2.11				
			OpenFF-2.0.0				
TYK2	66,425	Amber14sb	Espaloma-0.3.1	13	2H_2_O	3.47	Hahn et al.^30^
			GAFF-2.11				
			OpenFF-2.0.0				
Thrombin	49,471	Amber14sb	Espaloma-0.3.1	23	3H_2_O	5.87	Hahn et al.^30^
			GAFF-2.11				
			OpenFF-2.0.0				
MCL1	34,829	Amber14sb	Espaloma-0.3.1	25	none	4.19	Hahn et al.^30^
			GAFF-2.11				
			OpenFF-2.0.0				
Cyclophilin D	31,861	Amber99sb-ildn	Espaloma-0.3.1	10	none	8.49	Alibay et al.,^5^ Ries et al.^52^
			GAFF-2.11				
			OpenFF-2.0.0				
SAMPL6-OA	9,204	Espaloma-0.3.1	Espaloma-0.3.1	7	none	3.79	Rizzi et al.,^85^ Isik et al.^86^

We computed binding affinities for these 139 receptor–ligand pairs in triplicate using FEP or MM­(PB/GB)­SA, except for the cyclophilin D set, where five FEP replicates were used instead. For all protein targets, we computed binding affinities using three popular small-molecule force fields: GAFF-2.11,^65^ OpenFF-2.0.0,^66^ and Espaloma-0.3.1.^67^ For the SAMPL6-OA set, only Espaloma-0.3.1 was used.^67^ Cyclophilin D was described with the Amber99sb-ildn force field to allow comparison with previous studies.^5,52,53^ Among our MM­(PB/GB)­SA calculations, we compared results from the Poisson–Boltzmann model with results from the generalized Born model and, for each solvation model, evaluated the effects from using either no entropy contribution or using the IE or C2 entropy contribution. Together, our validation sets comprise 1,269 FEP and 7,254 MM­(PB/GB)­SA calculations.

We quantified the agreement between calculated Δ*G*_calc_ and experimental Δ*G*_exp_ affinities using the Pearson ρ, Kendall τ, and Spearman *r*_S_ correlation coefficients. Here, Pearson ρ quantifies the linear correlation between calculated and experimental values, accounting for magnitudes rather than just ranks, although it remains sensitive to outliers. In contrast, Kendall τ and Spearman *r*_S_ quantify agreement in ranking and are far less sensitive to outliers. Whereas Spearman *r*_S_ quantifies whether the ligand ranking according to Δ*G*_exp_ agrees with the ligand ranking according to Δ*G*_calc_, Kendall τ quantifies the concordance between pairs of ligands. Upon comparison of correlation coefficients from different receptors, it is critical to note that Pearson ρ is highly sensitive to the dynamic range among the ligands, whereas Kendall τ and Spearman *r*_*S*_ are less sensitive to the dynamic range (Table 1, seventh column). In this study, we strongly rely on Kendall τ as a key quality measure for Δ*G*_bind_ calculations since it has been shown to be a robust estimator,^88^ while reporting Pearson ρ and Spearman *r*_S_ as complementary measures. In addition to the correlation coefficients, we computed the RMSE, mean signed error (MSE), and mean unsigned error (MUE) of Δ*G*_calc_ relative to Δ*G*_exp_ (see the Supporting Information methods). To focus on the global correlation between Δ*G*_calc_ and Δ*G*_exp_ across all receptor–ligand pairs, we furthermore subtracted from each set its corresponding MSE from Δ*G*_calc_ to yield offset-corrected Δ*G*_calc_^oc^ values and, respectively, offset-corrected RMSE (ocRMSE, see the Methods section). Thereby, ocRMSE ignores systematic offsets between Δ*G*_calc_ and Δ*G*_exp_, for instance, owing to insufficient sampling of the apo state or owing to a systematic bias in receptor–ligand interactions as modeled by the force field. ocRMSE values may be compared with results from RBFE calculations since the latter are blind to such systematic offsets. Uncertainties of the statistical measures were derived by bootstrapping among the ligands for each set. Thus, critically, the errors bars for statistical measures are not only caused by limited sampling but furthermore stem from the limited number of 7 to 29 ligands per receptor.

### Comparison of FEP Results with Experiments

Figure 4 presents correlation plots between calculated affinities Δ*G*_calc_ obtained with GAFF and experimental affinities Δ*G*_exp_. Values are listed either for each of the seven protein targets individually (small panels) or for all seven targets combined (large panel). The aggregated plot displays the data after applying set-specific offset corrections. Insets report Pearson ρ, Kendall τ, MSE, and RMSE for each data set, as well as ocRMSE for the aggregated data. Correlation plots obtained with OpenFF or Espaloma are shown in Figures S2 and S3. Notably, we removed a single extreme outlier given by the binding of ligand lig_4 to thrombin (Figure S20) from the statistical quality measures to avoid a bias from a single receptor–ligand pair (see Figure 5). Lig_4 comprises a cationic amidinium moiety (−C–(NH_2_)_2_^+^) forming a salt bridge to Asp-189 of thrombin (Figure S24). We speculate that the greatly overestimated affinity of lig_4 may be a consequence of an overly stable amidinium–carboxylate bridge modeled by the three force fields.

**Figure 4:**
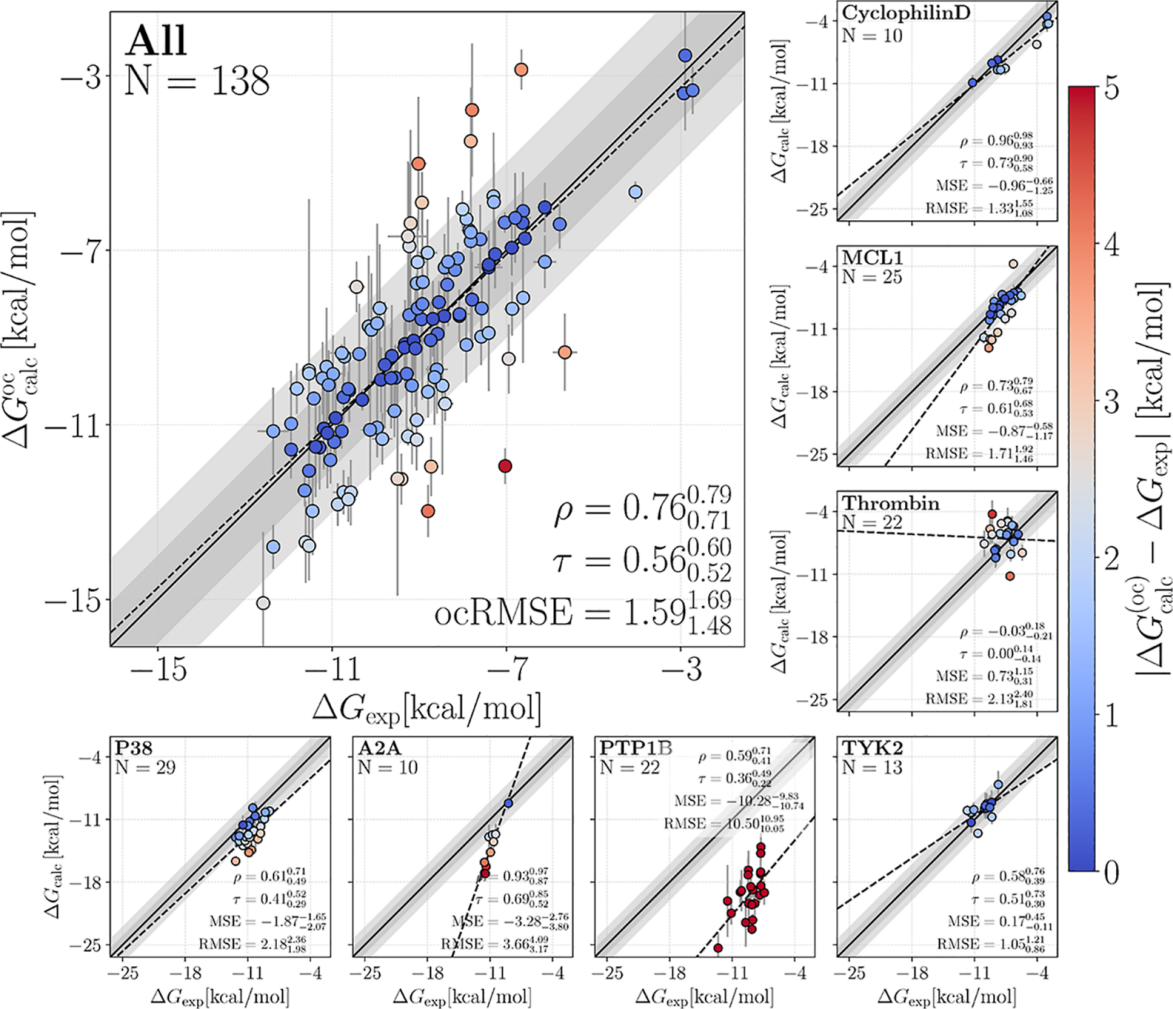
Calculated affinities Δ*G*_calc_ (or offset-corrected calculated affinities, Δ*G*_calc_^oc^) versus experimental affinities Δ*G*_exp_ from FEP with GAFF-2.11. Results show Δ*G*_calc_ for individual set (small panels, see labels for protein name and number of ligands *N*) and Δ*G*_calc_^oc^ collected from all sets (large panel). Insets show Pearson ρ, Kendall τ, MSE, and RMSE (or ocRMSE)—the last two in kcal mol^–1^—for each data set with their corresponding 68% confident interval. Colors of dots indicate the absolute deviation between Δ*G*_calc_ (or Δ*G*_calc_^oc^) and Δ*G*_exp_ (color bar). Dark and light gray diagonal regions indicate 1 or 2 kcal mol^–1^ deviations, respectively. Dashed lines are linear fits shown to guide the eye. A single outlier (lig_4, thrombin) has been removed. Error bars show uncertainties obtained via three independent replicates. Figure style has been inspired by ref 87.

**Figure 5:**
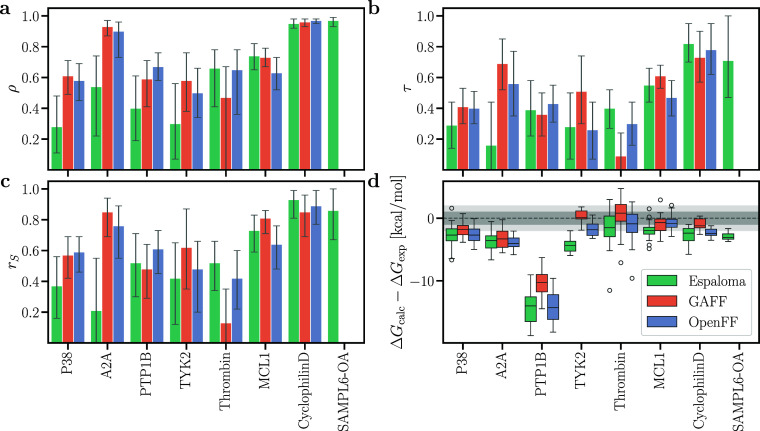
(a) Pearson ρ, (b) Kendall τ, (c) Spearman *r*_S_, and (d) deviations between calculated and experimental binding free energies Δ*G*_calc_ – Δ*G*_exp_ from FEP using Espaloma-0.3.1 (green), GAFF-2.11 (orange), and OpenFF-2.0.0 (blue). Results are shown for seven different data sets (labels at abscissa). Error bars represent the 68% confident interval obtained from bootstrapping among the ligands from each set. For Δ*G*_calc_ – Δ*G*_exp_, dark and light gray horizontal regions indicate 1 or 2 kcal mol^–1^ deviations, respectively. Box plots present the median (50th percentile) as a line within the box, and the lower and upper box edges correspond to the first (25th percentile) and third (75th percentile) quartiles, respectively. Whiskers extend to the smallest and largest data points within the 1.5 × interquartile range (IQR = Q3 – Q1), while outliers beyond this range are shown as circles.

For most receptors and force fields, the affinities were overestimated, as shown by mostly negative MSE values between typically −4 and 0 kcal mol^–1^ (small panels of Figures 4, S2, and S3; see also Figure 5d and Table S1). Such negative MSE values have been rationalized by insufficient sampling of the apo state, thereby partly missing the free energy cost of the induced-fit conformations transition upon ligand binding.^47^ An extreme case was PTP1B with MSE values between −14 and −10 kcal mol^–1^, pointing to a major free energy cost for the induced-fit conformational transition that was not captured during FEP simulation, in line with the value of approximately −10 kcal mol^–1^ reported by Chen et al.^47^

Statistical quality measures for the agreement with experiments greatly depended on the protein target and the force field, with RMSE values far below 2 kcal mol^–1^ for cyclophilin D with GAFF up to approximately 14 kcal mol^–1^ for PTP1B with Espaloma and OpenFF These variations were likewise reflected by Kendall τ, which spanned 0.82 for cyclophilin D with Espaloma down to poor τ values for thrombin. Pearson ρ and MSE values reported similar trends. The wide range of correlations obtained with different receptors and different force fields are summarized by Figure 5 and Table S1.

Each reported binding free energy corresponds to the average across three independent replicas (five for cyclophilin D), with uncertainties represented by the standard error of the mean (SEM). For systems such as MCL1 and P38, SEM values are small—typically in the range 0.5 to 1 kcal mol^–1^, as visible from the small error bars in the small panels of Figure 4. In contrast, PTP1B shows markedly larger SEMs, reflecting convergence challenges likely caused by occasional sampling of apo-like conformations. This highlights both the difficulty of capturing the induced-fit transition in PTP1B within typical simulation times and the utility of replica-based SEMs for revealing such uncertainties.

### Comparison of FEP Results from BindFlow with the Literature

Ross et al.^89^ evaluated the reproducibility of experimental RBFE values and reported an overall RMSE (weighted by number of ligands on each set) of 0.91 kcal mol^–1^ (95% CI: [0.83, 1.11]) and a weighted average Kendall τ = 0.71 (95% CI: [0.65, 0.74]). Thus, upon comparison of Δ*G*_calc_ with Δ*G*_exp_ in this study, the considerable intrinsic uncertainty of Δ*G*_exp_ must be kept in mind. Accordingly, an RMSE of ∼1 kcal mol^–1^ and a Kendall τ of ∼0.7 between Δ*G*_calc_ and Δ*G*_exp_ would indicate excellent agreement.

In addition, Ross et al.^89^ validated FEP+ software for RBFE calculations in combination with the OPLS4 force field^90^ against protein–ligand sets different from the ones used here. Beyond the differences in validation sets, their study employed RBFEs, whereas we performed ABFEs, which are generally more challenging. For these reasons, a direct, quantitative comparison of the RMSE and Kendall τ is not meaningful. Nevertheless, placing the results side by side helps contextualize the current state of the art in FEP calculations. FEP+ achieved an RMSE of 1.25 (95% CI: [1.17, 1.33]) and a Kendall τ of 0.51 (95% CI: [0.48, 0.55]). With Espaloma, GAFF, and OpenFF, we obtained slightly higher ocRMSE values, which may indicate that the closed-source OPLS4 force field^90^ performs somewhat better than the currently freely available force fields. However, the Kendall τ values obtained here were even slightly higher than those from FEP+, suggesting that our ABFE calculations are competitive in terms of ligand ranking.

Next, we systematically compared ABFE results from BindFlow with FEP+ by restricting the analysis to ligands that were present in both the data set of Chen et al.^47^ and our BindFlow simulations, resulting in 89 ligands across the P38, PTP1B, TYK2, and MCL1 sets. Both BindFlow and FEP+ systematically overestimated affinities for PTP1B (Figure S7, top row). Thus, to enable a quantitative comparison of ABFE values, we removed PTP1B from the data sets and focused the comparison on the remaining 67 ligands. FEP+ yielded systematically stronger binding affinities on these three sets compared to BindFlow, as quantified by the MSE of −4.29_–4.42_^–4.16^ kcal mol^–1^ for FEP+, versus −2.74_–2.95_^–2.53^, −1.10_–1.28_^–0.93^, and −1.79_–1.98_^–1.62^ kcal mol^–1^ for Espaloma, GAFF, and OpenFF, respectively (Figure S7, middle row). Keeping the PTP1B set and removing the constant offset for each set by subtracting the set-specific MSE from Δ*G*_calc_, FEP+ yielded considerably lower ocRMSE values compared to BindFlow when used with any of the three force fields. FEP+ also yielded higher correlation coefficients (Figure S7, bottom row). Thus, in light of these data, FEP+ may achieve superior ranking at the cost of overestimating the absolute binding affinity. We speculate that the superior ranking by FEP+ is primarily a consequence of using the OPLS4 force field, although alternative sampling algorithms may also play a role. Within this subset of ligands, GAFF achieved better agreement than Espaloma and OpenFF with experimental data, with ocRMSE = 1.52_1.40_^1.64^ kcal mol^–1^ and τ = 0.53_0.48_^0.58^ (Figure S7, bottom row).

Deflorian et al.^13^ reported RBFE calculations for the A2A system using FEP+ software. We converted their RBFE values into ABFEs by referencing the experimental binding affinity of ligand 4k and correcting for the MSE. From their data, we obtained ocRMSE = 0.65_0.47_^0.79^ kcal mol^–1^ and τ = 0.78_0.65_^0.90^. Evidently, since all ligands were similarly composed of three aromatic rings (Figure S17), RBFE required relatively small perturbations, rationalizing the high τ and exceptionally low ocRMSE. Although our ABFE calculations involved by far larger perturbations, we obtained decent agreement (Figure S10) with GAFF (ocRMSE: 1.64_1.35_^1.87^ kcal mol^–1^, τ = 0.69_0.52_^0.85^, Figure 4) and with OpenFF (ocRMSE: 1.02_0.80_^1.19^ kcal mol^–1^, τ = 0.56_0.33_^0.76^, Figure S2). Only with Espaloma, we obtained a rather poor τ of only 0.16_‑0.12_^0.44^ (Figure S3), which might indicate inaccurate modeling of the ligand interactions with the Na^+^ cofactor inside the A2A binding pocket.

The cyclophilin D set was first introduced by Alibay et al.^5^ for ABFE benchmarking and was recently employed to validate ABFE_workflow^52^ and A3FE.^53^ Excellent agreement with experiments was found across the three pipelines—ABFE_workflow, A3FE, and BindFlow—underscoring that the cyclophilin D set was the least challenging receptor–ligand set considered in this study (Figures S8 and 5).

The P38 and TYK2 systems have been used by several authors for validating binding free energy calculations using different methodologies.^17,47,49,51,87^ Figure S9 compares results from BindFlow with previous studies.^17,47,49,51,87^ Here, Lin et al.^17^ and Chen et al.^47^ achieved the best ranking of ligands, though at the cost of overestimating the absolute binding free energies, as shown by large negative MSE. Among our results, GAFF achieved comparable ranking at a less negative MSE.

Taken together, the reasonable agreement with previous data validates BindFlow’s FEP pipeline and demonstrates competitive agreement with experimental data for a wide range of ligands and receptors.

### Comparison of MM­(PB/GB)­SA with Experiments

MM­(PB/GB)­SA provides a computationally efficient alternative to FEP. Specifically, the MM­(PB/GB)­SA pipeline used here employed only ∼3 ns of MD simulations, corresponding to a 75-fold lower computing cost compared to the FEP pipeline used here. However, whereas the MMGBSA calculation from the MD frames is highly efficient, the MMPBSA calculation may take considerable additional computing time owing to the cost of Poisson–Boltzmann calculations. As expected from previous studies,^10^ absolute binding free energies obtained with MMGBSA (Figures 6d, S11d and S12d) or MMPBSA (Figures S13d, S14d, and S15d) revealed poor agreement with experimental values. For instance, MMGBSA without entropy contribution overestimated the absolute binding affinities to protein targets by approximately 25 to 75 kcal mol^–1^ (Figure 6d). Even upon correcting the binding affinity with a target-specific offset, ocRMSE values remained large (Figures S4–S6).

**Figure 6:**
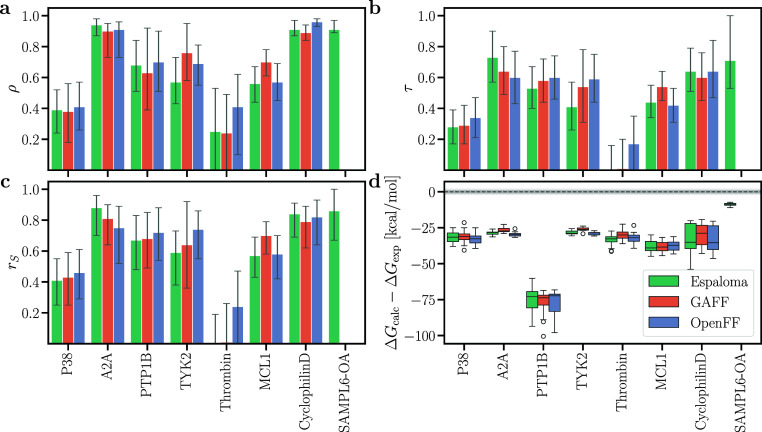
(a) Pearson ρ, (b) Kendall τ, (c) Spearman *r*_*S*_, and (d) deviations between calculated and experimental binding free energies Δ*G*_calc_ – Δ*G*_exp_ from MMGBSA results without entropy contribution. Presentation style according to Figure 5.

However, MMGBSA without entropy contribution achieved overall excellent Pearson, Kendall, and Spearman correlation coefficients for A2A, cyclophilin D, and SAMPL6-OA while still reaching good correlation coefficients for PTP1B, TYK2, and MCL1 (Figures S4–S6, and 6a–c). In contrast, correlation coefficients for P38 or thrombin were rather poor. The three force fields GAFF, OpenFF, and Espaloma achieved similar ranking. Thus, while MMGBSA is not suitable for obtaining absolute binding free energies (Figure 6d), it may provide for certain receptors reasonable ranking among ligands and, thereby, serve as a useful tool for screening large data sets of potential binders.

Using the Poisson–Boltzmann instead of the generalized Born solvation model had only moderate effects on the correlation coefficients (compare Figures S13 with [Fig fig6]), suggesting that the computationally more efficient generalized Born model yields a good starting point for computational studies. Using the IE entropy contribution had only a small effect on the correlation coefficients (compare Figure S15 with S12), whereas the C2 entropy contribution decreased the correlation coefficients (compare Figure S14 with S11). Hence, in line with previous findings,^91^ entropy contributions should be used with care or tested for the receptor of interest because entropy contributions may deteriorate the ranking by MM­(PB/GB)­SA.

### Ligand Ranking by FEP versus MMGBSA and by Different Force Fields

Figure 7 summarizes the overall performance of FEP versus MMGBSA among the three force fields, as collected from all receptor–ligand pairs, thus including easy receptors such as cyclophilin D and challenging receptors such as thrombin (see also Table S1). Here, the matrices in Figure 7b/d visualize whether one combination of method/force field, such as FEP/Espaloma, outperforms another combination, such as MMGBSA/GAFF (see the Methods section for the applied significance test). The following conclusions are drawn from the analysis: (i) In terms of ocRMSE, FEP by far outperformed MMGBSA (Figure 7c/d). These findings suggest that errors caused by major approximations underlying MMGBSA remain after correction of Δ*G*_calc_ by the MSE. (ii) In terms of ranking by Kendall τ, FEP overall outperformed MMGBSA (Figure 7a/b). An exception was given by FEP/Espaloma that showed only insignificant differences relative to those of MMGBSA (Figure 7b). (iii) For a given method (FEP or MMGBSA), the three force fields showed no statistically significant differences (Figure 7b/d), except among the poor ocRMSE by MMGBSA. Thus, simulations with additional ligands will be required to test whether the slightly lower τ and slightly larger ocRMSE obtained with Espaloma are significant or whether they were caused by the limited set of receptor–ligand pairs considered in this study.

**Figure 7:**
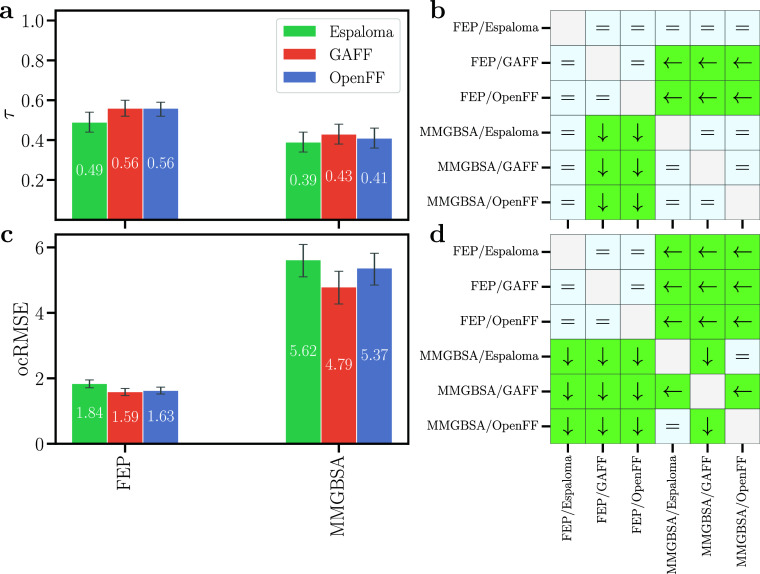
Summary of agreement between FEP or MMGBSA calculations and experimental data quantified by (a/b) Kendall τ and (b/c) offset-corrected root-mean-squared error (ocRMSE) in kcal mol^–1^, summarized from all receptors. Error bars show 68% confident intervals obtained from bootstrapping among the ligands from all sets. Pairwise significance difference matrices are for (b) τ and (d) ocRMSE. An arrow indicates a statistically significant difference pointing to the combination of method/force field with the better statistical metric. Equal symbols indicate an insignificant difference. The set SAMPL6-OA is excluded to ensure consistence in the analysis across force fields. The extreme outlier lig_4 from the thrombin set was excluded.

### Computational Costs

BindFlow’s completion time mostly depends on (i) the aggregated MD simulation time, as defined by the number of ligands and replicas, simulation times, and number of λ-windows for FEP or samples for MM­(PB/GB)­SA, together with (ii) the performance of the GROMACS MD engine, which depends on parameters such as hardware architecture, system size, or integration time step.

By modeling BindFlow execution within a queue-based scheduling environment similar to SLURM,^77,92^ we estimated completion times for common multiligand campaigns. Details are presented in the Performance Section of the BindFlow online documentation. For example, for the thrombin system and the protocol described in this study, a cluster with 200 Nvidia RTX A4000 GPUs, each equipped with 10 CPU cores of Ryzen Threadripper PRO 3975WX, would complete 580 FEP or 39,965 MMGBSA calculations within 1 week. On average, MMGBSA completed in a 75-fold shorter time than FEP.

However, these values may vary considerably upon using alternative scheduling strategies, for instance, involving more λ-windows in combination with shorter simulation times per window, or by adhering to nonlinear instead of equidistant λ-window spacing.^93^ In addition, we here used relatively large water compartments by keeping a distance of 1.5 nm between the receptor and simulation box surface, suggesting that tuning the number of water molecules may provide a route for optimizing the computational cost in future campaigns.

## Discussion

We introduced BindFlow, a free and user-friendly software program for ABFE calculations. BindFlow offers fully automated default pipelines for FEP and MM­(PB/GB)­SA, requiring minimal user intervention while providing extensive configuration options for advanced users. Comprehensive online tutorials and documentation support accessibility. The software is developed according to best coding practices, providing transparency, reproducibility, and a solid foundation for future community-driven development. By scheduling its tasks with Snakemake, BindFlow optimizes the use of distributed platforms, is resilient to rare failures of individual simulations, and enables seamless interruption and continuation of large-scale multiligand campaigns. It supports globular and membrane proteins as well as nonprotein host–guest systems, provided that a GROMACS-compatible topology can be generated. Thereby, BindFlow integrates each of the six suggested properties for ABFE solutions, as outlined in the Introduction section, into a single workflow (see also the Supporting Discussion).

We validated BindFlow by computing Δ*G*_bind_ values for 139 ligands binding to one of eight different receptors using FEP together with MBAR or using MM­(PB/GB)­SA. Overall, we found that BindFlow yields competitive Δ*G*_bind_ values compared to the standards in the field. As expected from previous studies, the accuracy of Δ*G*_bind_ values and ligand ranking greatly depend on the target and the chemical moieties of the specific ligand.

For the 139 receptor–ligand pairs, in terms of accuracy of Δ*G*_bind_ predictions quantified by ocRMSE, FEP by far outperformed MM­(PB/GB)­SA. In terms of ligand ranking, FEP still outperformed MMGBSA significantly; however, for several receptors studied here, ranking by MMGBSA was remarkably good. Only for P38 and thrombin did MMGBSA achieve poor ranking. Thus, our study underscores that MMGBSA may provide a computationally inexpensive method for prescreening large sets of ligands prior to validation by the more expensive FEP. However, future studies will need to establish whether favorable correlations between MM­(PB/GB)­SA and experiments, as observed here, also hold for noncongeneric sets of ligands.

Beyond the benchmark simulations presented here, BindFlow has recently been applied successfully for combining ∼60,000 MM­(PB/GB)­SA calculations with Bayesian active learning;^42^ for interpreting X-ray crystallographic data from the antiviral drug tecovirimat binding to the phospholipase F13 from monkeypox virus;^44^ and as a module to automate the system preparation phase of a typical MD simulation.^94^ These use cases highlight that BindFlow is ready for production use.

BindFlow will be useful to systematically test and improve small-molecule force fields. For instance, we spotted that the affinity of lig_4 binding to thrombin was greatly overestimated by 7 to 13 kcal mol^–1^, possibly caused by an overly strong amidinium–aspartate salt bridge (Figure S24). This interpretation is supported by the fact that larger partial charges used by Espaloma correlated to an even stronger computed affinity. Since amidinium moieties are quite common in drugs, these findings point toward options for further force field refinements.

Future implementations may further enhance the capabilities of BindFlow. (i) Enhanced sampling techniques, such as Hamiltonian replica exchange,^95–97^ may accelerate convergence and, thereby, reduce the computational cost. Enhanced sampling may, specifically, improve the sampling of the apo state and lead to smaller MSE values. (ii) The adaptive allocation of computational resources may focus sampling to λ windows with increased sampling challenges.^53^ (iii) Charged ligand systems (e.g., MCL1, thrombin, and PTP1B) showed satisfactory accuracy in our validations. However, finite-size effects in charge-imbalanced systems, primarily due to the use of particle-mesh Ewald method,^98,99^ remain a concern.^100,101^ Charge corrections^102,103^ such as the coalchemical ion approach^12^ represent viable solutions to address this issue. (iv) Water exchange between the binding site and bulk solvent represents a slow sampling process that is difficult to capture during the short MD simulations performed in FEP.^12^ Monte Carlo-based water swap methods in a grand canonical ensemble,^104,105^ methods based on inhomogeneous fluid solvation theory,^106^ or specialized hydration-shell generators such as SOLVATE^78,107^ have shown promise in treating the explicit water. (v) Additional flavors of end-point free energy methods, as alternative to the single-trajectory MM­(PB/GB)­SA approach used here, may be readily implemented, for instance, involving three-trajectory techniques, quantum corrections,^108^ or central limit free energy perturbation.^109^ Future versions of BindFlow may include such methodologies.

## Conclusions

We presented BindFlow, an open-source and user-friendly pipeline for ABFE calculations that integrates both rigorous FEP and efficient end-point methods [MM­(PB/GB)­SA] into a single unified framework. Validation on 139 receptor–ligand pairs showed that BindFlow achieves predictive performance comparable to standards in the field while offering full automation and extensive customization. FEP achieved more accurate Δ*G*_bind_ predictions and ligand ranking than MM­(PB/GB)­SA. However, MM­(PB/GB)­SA provided remarkable ligand rankings for several systems at a fraction of the computational cost, highlighting its value for large-scale computational prescreening campaigns. Thus, we anticipated that the combination of MM­(PB/GB)­SA and FEP will be powerful for balancing efficiency and accuracy in future BindFlow applications. BindFlow scales efficiently across HPC environments, enabling high-throughput calculations with an excellent resource utilization. BindFlow is designed to serve both nonexpert and expert users: it provides a straightforward, plug-and-play interface with extensive online documentations and tutorials that allows users to perform ABFE calculations without detailed technical knowledge, while also offering advanced configuration options for expert users who wish to customize specific aspects of the workflow. By combining automation, flexibility, accessibility, documentation, and HPC-oriented scalability, BindFlow lowers the entry barrier for routine ABFE calculations and provides a platform for modern drug discovery or for systematically improving small-molecule force fields.

## Methods

### Building Simulation Systems

BindFlow version 0.13.post24 was used. Simulations followed largely the current default settings of BindFlow. The setup protocols are fully automated yet may be easily adapted by the user via the *global_config* keyword, which is a Python dictionary. This dictionary may be conveniently defined with a YAML file (Listing S7). The simulations presented here were set up as follows.

The task *Build Simulation System* (Figure 3) assembles the simulation systems and defines force fields. Starting structures were taken from previous studies.^5,13,30,52,85,86^ Soluble proteins, SAMPL6-OA, and ligands were placed in an octahedral simulation box, keeping a distance of 1.5 nm between the solute and box boundary. The systems were solvated by explicit water with the GROMACS solvate module and approximately 150 mM NaCl was added, thereby neutralizing the system. A minimum distance of 1 nm between ions and nonsolvent molecules was used. The structure of the membrane protein A2A was taken from ref 13. BindFlow does not build simulation systems of membrane proteins. Thus, the A2A system was built using CHARMM-GUI^110^ by placing the protein in a simulation box of a hexagonal prism and by embedding it in a membrane of 172 1-palmitoyl-2-oleoyl-*sn*-glycero-3-phosphocholine (POPC) lipids.

Ligand interactions were described with Espaloma-0.3.1,^67^ GAFF-2.11,^65^ or OpenFF-2.0.0.^66^ Ligand topologies were generated within BindFlow using the TOFF Python library.^83^ Cyclophilin D was described with Amber99sb-ildn,^80^ as used by previous studies.^5,52,53^ All other proteins were described with Amber14sb,^111^ using OpenMM^112^ and ParmEd^113^ for generating force field topologies. The host molecule octa acid of the SAMPL6-OA system was described with Espaloma-0.3.1,^67^ and the topologies were generated with TOFF.^83^ Water was modeled with the TIP3P model,^82^ and ion parameters were taken from the definitions provided with Amber99sb-ildn.^80^

### Simulation Parameters

The tasks *Setup Complex Equilibration*, *Setup Ligand Equilibration*, *Setup Complex FEP*, *Setup Ligand FEP*, and *Setup Complex MM­(PB*/*GB)­SA* (Figure 3) define MD parameters and set up directory structures for the initial equilibration, FEP, or MM­(PB/GB)­SA simulations.

All simulations were carried out with GROMACS, version 2022.4.^76^ The geometry of water acting as a cofactor was constrained with LINCS,^114^ while the geometry of all other water molecules was constrained with SETTLE.^115^ All other bonds were constrained with LINCS^114^ if not stated otherwise. Hydrogen mass repartitioning was used with a mass repartition factor of 2.5. During production simulations, a time step of 4 fs was used. Dispersive interactions and short-range repulsion were modeled by using a Lennard-Jones potential with a 1 nm cutoff. Electrostatic interactions were calculated with the particle-mesh Ewald^98,99^ method, applying a real-space cutoff of 1 nm.

The temperature was maintained at 298.15 K. For the initial equilibration of membrane protein–ligand complexes, the temperature was maintained using velocity rescaling (τ_t_ = 1 ps). Here, the protein and Na^+^ cofactor were coupled to the same thermostat. During all other simulations, the temperature was maintained by using Langevin dynamics (τ_t_ = 2 ps). The pressure was maintained at 1 bar for membrane protein–ligand complexes using semi-isotropic stochastic cell rescaling^116^ with a time constant of τ_p_ = 5 ps during the initial equilibration. The same barostat was used during the perturbation phase for membrane protein–ligand complexes, but the pressure was maintained at 1 atm (1.01325 bar) and τ_p_ = 2 ps was used. For all other simulations, the pressure was controlled at 1 atm with the isotropic Berendsen barostat during equilibration (τ_t_ = 1 ps)^117^ and the Parrinello–Rahman barostat during production (τ_p_ = 2 ps).^118^

### Initial Multistep Equilibration Protocol

After minimizing the energy with the steepest-descent algorithm, each system was equilibrated with a multistep protocol (see Supporting Information methods).

### FEP Calculations

Following Alibay et al.^5^ and Ries et al.,^52^ Δ*G*_bind_ from FEP was computed according the thermodynamic cycle shown in Figure 2b. Coulomb interactions of the ligand in solvent were decoupled over 11 λ points (Figure 2b, transition 1 → 2), followed by decoupling of Lennard-Jones interactions over 11 λ points (transition 2 → 3). Both, inter- and intramolecular interactions were decoupled. A 10 ns equilibrium simulation together with the software MDRestraintsGenerator^119^ was used to obtain the optimal Boresch restraints,^39^ the free energy cost for introducing restraints to the ligand in solvent (transition 3 → 4), and the initial frame for the complex decoupling simulations. Inter- and intramolecular Lennard-Jones interactions of the ligand in the receptor were activated over 21 λ points (transition 5 → 6), followed by 11 λ points for activating the Coulomb interactions (transition 6 → 7). Finally, Boresch restraints were removed for the ligand in the receptor over 11 λ points (transition 7 → 8).

For each λ window, the system was energy minimized and equilibrated with a three-step protocol (see Supporting Information methods). Each window was simulated for 10 ns using a stochastic dynamics integrator. During simulations that decouple Lennard-Jones interactions, a soft-core potential (α = 0.5, σ = 0.3, power = 1) was used. Free energy differences were computed with MBAR.^63^ As a control, free energies were additionally computed using TI.^68^ BindFlow reports a warning if results from MBAR and TI differ by more than 0.5 kcal mol^–1^ as such differences may indicate poor convergence or simulation instabilities. Alchemlyb-2.0.0 was used for MBAR and TI evaluations.^120^

The binding free energy was computed via (Figure 2B)

6 $$\begin{array}{ll} \Delta G_{bind}= & \Delta G_{coul}^{solv,dcpl}+\Delta G_{vdw}^{solv,dcpl}+\Delta G_{rest}^{solv,on}\\ & +\Delta G_{vdw}^{rcp,cpl}+\Delta G_{coul}^{rcp,cpl}+\Delta G_{rest}^{rcp,off} \end{array}$$

Here, Δ*G*_coul_^solv,dcpl^ and Δ*G*_vdw_^solv,dcpl^ denote free energies for decoupling (dcpl) Coulomb and Lennard-Jones interactions of the ligand in solvent (solv), respectively. Δ*G*_rest_^solv,on^ denotes the free energy for turning on Boresch restraints (rest) for the ligand in solvent, which is computed analytically. Here, Δ*G*_rest_^solv,on^ is defined such that the decoupled state of the ligand in solvent corresponds to the standard state with a ligand concentration of 1 mol/L.^39,119^ Δ*G*_vdw_^rcp,cpl^ and Δ*G*_coul_^rcp,cpl^ denote the free energy for activating (coupling, cpl) Lennard-Jones and Coulomb interactions for the ligand in the receptor (rcp), respectively. Δ*G*_rest_^rcp,off^ denotes the free energy cost for turning off the Boresch restraints for the ligand in the receptor.

BindFlow reports the statistical error obtained from MBAR^63^ together with Gaussian error propagation. However, owing to autocorrelations, this value greatly underestimates the true uncertainty of Δ*G*_bind_. Thus, we carried out the whole pipeline including setup, equilibration, and FEP in three (five for Cyclophilin D^5^) independent replicates and report the respective SEM as error bars in the correlation plots.

### MM­(PB/GB)­SA Calculations

The initial multistep equilibration for MM­(PB/GB)­SA calculation was carried out as described in the Supporting Information methods. Following Su et al.,^91^ MM­(PB/GB)­SA values were computed from multiple short simulations, rather than from a single long simulation. Accordingly, 20 starting frames were taken from a 950 ps equilibrium simulation, using one frame every 50 ps. From each frame, a 100 ps simulation was carried out by writing a frame every 5 ps. Here, we follow Genheden and Ryde,^10^ who recommended using an output frequency of 1 to 10 ps. MM­(PB/GB)­SA values were computed with the gmx_MMPBSA software,^69^ yielding 20 samples of the binding affinity. Cofactors were defined as a part of the receptor for the MM­(PB/GB)­SA calculation. For each complex, the pipeline was run in three independent replicates. Binding affinities reported here were computed by averaging over 60 samples from the three replicates with 20 samples per replica, and the error was derived as the SEM and reported as error bars in correlation plots. PB and GB models were used, each without an entropy contribution or using the IE or C2 entropy contribution. The main text reports results from the GB model without entropy contribution, while all other results are provided in the Supporting Information.

## Supplementary Material



## Data Availability

BindFlow is free software published under the GPL-3.0 license. The code is currently hosted at https://github.com/ale94mleon/BindFlow. Documentation is available at https://bindflow.readthedocs.io. Scripts and input files required for reproducing our results and analysis are accessible at: https://github.com/ale94mleon/bindflow-api-paper-si.

## Notes

Supporting Information includes the following: supporting methods and discussion; example code for an MM­(PB/GB)­SA calculation of a membrane system using BindFlow (Listing S1); examples of optional dictionaries used to define system components in BindFlow (Listing S2–S6); list of user-configurable options passed to *global_config* (Listing S7); disk space usage of BindFlow simulations for FEP and MM­(PB/GB)­SA workflows, including effects of data compression (Figure S1); correlation plots for FEP using OpenFF or Espaloma force fields and for MMGBSA without entropy contribution using GAFF, OpenFF, or Espaloma force fields (Figures S2–S6); comparison of BindFlow FEP results with literature data (Figure S7–S10); statistical metrics for MMGBSA with C2 or IE contribution (Figures S11 and S12); statistical metrics for MMPBSA with and without entropy contribution (Figures S13–S15); chemical structures of ligands with reported experimental affinities for all validation sets (Figures S16–S23); salt bridge interaction between lig_4 and Asp-189 in the thrombin complex (Figure S24); and summary table of agreement between calculated and experimental binding free energies across all validation sets (Table S1) (PDF)
